# Evaluation of the Human Interference on the Microbial Diversity of Poyang Lake Using High-Throughput Sequencing Analyses

**DOI:** 10.3390/ijerph16214218

**Published:** 2019-10-30

**Authors:** Haiming Qin, Lanyue Cui, Xinyi Cao, Qian Lv, Tingtao Chen

**Affiliations:** 1 Key Laboratory of Poyang Lake Environment and Resource Utilization, Ministry of Education, Nanchang University, Nanchang 330031, China; qinhaiming@qfnu.edu.cn (H.Q.); cuilanyueliang@163.com (L.C.); 18062370328@163.com (X.C.); lvqianncu@yeah.net (Q.L.); 2 School of Life Sciences, Qufu Normal University, Qufu 273165, China; 3 National Engineering Research Center for Bioengineering Drugs and the Technologies, Institute of Translational Medicine, Nanchang University, Nanchang 330031, China

**Keywords:** Poyang Lake watershed, bacteria, high-throughput sequencing, anthropogenic disturbance, water, sludge

## Abstract

The Poyang Lake Watershed (PLW) is regarded as an air temperature moderator, as well as a wind energy, food resources and good habitat in the Jiangxi Province, People’s Republic of China. However, with the increasing of anthropogenic disturbance on PLW, there are few studies focused on the effects of human activities on microbial composition in Poyang Lake. In the present study, a high-throughput sequencing method was used to identify the microbial composition in water and sludge in Dahuchi (DHC, sub-lake of Poyang Lake National Nature Reserve), Shahu (SH, sub-lake of Poyang Lake National Nature Reserve), Nanhu (NH, sub-lake out of Poyang Lake National Nature Reserve), Zhelinhu (ZLH, artificial reservoir), Sixiahu (SXH, sub-lake artificially isolated from Poyang Lake) and Qianhu (QH, urban lake). Results of the present study illustrated the various bacterial diversity between different lakes, for example, at the phylum level, *Actinobacteria* and *Cyanobacteria* showed low abundance in water samples of ZLH and QH, and high abundance in DHC. In addition, anthropogenic disturbance and human activities decreased the abundance of probiotic bacteria (*Actinobacteria*, *Cyanobacteria, Chloroflexi* and *Acidobacteria*) and increased the abundance of pathogenic bacteria (*Acinetobacter*, *Aeromonas* and *Noviherbaspirillum*). The enrichment of pathogenic bacteria in polluted lakes, in turn, may cause potential threats to human health.

## 1. Introduction

Natural disasters, biogeochemical cycles, interaction between lakes and rivers and human activities, have great influences upon the environment of lake–river ecotone [1], of which human activities (e. g. impoundments and diversions to meet water, energy and transportation needs, fishing and breeding aquatics) have extensively altered the wetland ecosystem, harmed human health and toxified most plants and animals [2,3]. For example, the agricultural non-point source pollution is going to be the main factor of river pollution in China [4]; the high input of agriculture production has resulted in the surplus of nitrogen and phosphorus in farm fields, increased the content of nitrogen and phosphorus in surface waters, leading to eutrophication and instability of the aquatic ecosystems [5]. 

In addition, human activities can not only alter the natural environment and food web structures significantly [6], but also influence the wetland plant communities and wetland microbial communities deeply [7].

**Figure 1:**
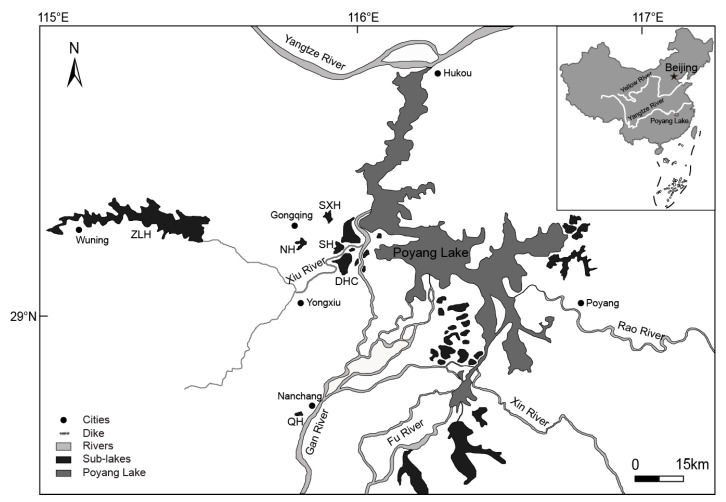
Distribution of Poyang Lake, sub-lakes and associated rivers in China.

Poyang Lake is the largest fresh water lake in China, and is also one of the world’s six major wetland systems, which is located in the floodplain of the middle and lower reaches of the Yangtze River, southeastern China (Figure 1) [8]. It belongs to the Poyang Lake Watershed (PLW), and has major responsibility for the protection of the natural habitat, rare migratory birds such as the Siberian crane, white stork, black stork, swan and white-napped crane, as well as other aquatic organisms [9]. PLW, as a unique and important ecosystem fed by the Gan River, Fu River, Xiu River, Rao River and Xin River [10], is regarded as an air temperature moderator, wind energy, food resources and good habitat, in Jiangxi Province [10,11]. This ecosystem acts as a wetland ecosystem occupying the interface between terrestrial and aquatic systems, which brings many benefits to society and gives support to coastal fisheries, habitat for wildlife, flood mitigation, protection from shoreline erosion, wastewater treatment, and water-quality enhancement [12]. It also has important ecological functions in nutrient cycling, energy flow, climate regulation, sediment accretion, pollutant filtration and biodiversity maintenance [13]. There are six studied lakes in the PLW: (Dahuchi (DHC), Shahu (SH), Nanhu (NH), Sixiahu (SXH), Zhelinhu (ZLH) and Qianhu (QH)): DHC, SH and NH are the sub-lakes of Poyang Lake, which act as the main habitat of wintering birds; SXH is losing hydrological features gradually because of the building of dams; ZLH is an artificial reservoir for generating electricity, possessing integrated functions of flood control, irrigation, and aquaculture; QH is an urban lake with a degree of eutrophication, often used for sanitary sewage discharging.

The community structure of the ecosystem is built on microorganisms [14]. Bacteria are essential in the aquatic carbon cycle, with the ability of disposing the organic carbon carried in running waters [15,16]. As one of the most important constituent elements of the wetland ecosystem, the microbial diversity is sensitive to environmental changes with many functions: It greatly affects the energy conversion, material recycling, nutrients and element transformation, accumulation and migration [1], for instance, soil microorganisms mediate nitrification, denitrification, and methanogenesis to regulate ecosystem process and feedback to influence the atmospheric chemistry [7,17]. 

So far, studies on bacterial diversity in the lakes of the PLW are still lacking. In the present study, a high-throughput sequencing method was applied to extensively and systematically investigate the bacterial diversity in the water and sludge of the sub-lakes of this PLW, which provides data to evaluate the effect of human activities on the microbial composition and the ecosystem of Poyang Lake.

Based upon the observation data, the objectives of this study were (1) to analyze the changes of bacterial communities and diversity in six studied lakes, (2) to evaluate the impact of anthropogenic disturbance on bacterial communities and diversity with spatial differences, and (3) to provide a scientific basis for PLW ecosystem health maintenance and biodiversity conservation.

## 2. Materials and Methods

### 2.1. Study Sites

There are six lakes we sampled in the Poyang Lake Watershed (PLW). Three of them are sub-lakes of Poyang Lake: (1) Dahuchi (DHC) (28°08′–29°16′ N and 115°91′–115°97′ E), (2) Shahu (SH) (29°16′–29°20′ N and 115°91′–115°95′ E) and (3) Nanhu (NH) (29°17′–29°23′ N and 115°79′–115°92′ E). DHC and SH are connected to the main lake area of the Poyang Lake by the Xiu River when the water level reaches 17.3 m; except for the fishing activities of local fishermen, there are few man-made disturbances. NH also connects to the main lake area of Poyang Lake when the water level exceeds 17.3 m. Some aquaculture ponds surround NH, and fishermen would go to the lake at different times to catch fish with customized nets with electricity, so human activities interfere greatly at NH. (4) Zhelinhu (ZLH) (29°19′–29°37′ N and 115°34′–115°53′ E), located at the upstream of the Xiu River, is a medium nutrient level lake. It is an artificial reservoir, which is mainly used for power generation and has integrated functions of flood control, irrigation, shipping and aquaculture. The maximum water depth is 45 m, the visibility is about 9 m, and the water quality of ZLH is good. (5) Sixiahu (SXH) (29°25′–29°30′ N and 115°87′–115°91′ E) is located at the northwest of Poyang Lake National Nature Reserve. It used to be a natural sub-lake of Poyang lake, while local fishermen built low dams in the late 1980s to catch fish. Around 2004, fishermen used nets to divide the lake into different areas for fish stocking, and no more fishing occurred in the dry season, so the fishery pattern of this sub-lake changed. At present, SXH has been seriously disturbed and has gradually lost the hydrological characteristics of the natural sub-lakes with a degree of eutrophication. (6) Qianhu (QH) (28°64′–28°66′ N and 115°80′–115°81′ E), a newly formed urban lake with a certain degree of eutrophication, is located in the southwest of Nanchang City, surrounded with several colleges, the administrative center of Jiangxi province, and the Qianhu Hotel.

### 2.2. Sampling

Sludge samples and water samples were collected from six lakes between 12 and 13 July 2018. Each lake was sampled in triplicate at the same time, and the sampling sites were chosen subjectively for their accessibility, and these embraced a range of morphometric (e.g., area, connectivity to adjacent river channel), hydrological and water quality variables [18].

Water samples were collected by a sterile sampler from 0.5 m below the surface of the lakes, and each sediment sample was taken as 500 g of topsoil with a flamed towel. Samples from different lakes were transported to the laboratory on ice within 2 h of collection and processed immediately. Each water sample was filtered through a 0.22 μm pore size filter (diameter 45 mm; Millipore, MA, USA), and the filters were stored at −80 °C for DNA extraction [3].

### 2.3. Extraction of Genome DNA and High-Throughput Sequencing

The sediment samples were diluted five-fold with ddH_2_O and homogenized with a bead-beating method. Samples were suspended in 1 mL lysis buffer (LBS) containing 0.3 g sterile zirconium beads in the screw-capped tube, and we bead-beat the tubes at 8000 rpm for 3 min in a mini-bead beater. The total genome DNA of each sample was extracted by a TIANamp Genomic DNA kit (TIANGEN) combined with the bead-beating method, as previously described. 

After this, the genomic DNA was sent to a high-throughput sequencing company (Biomarker Technologies Corporation, Beijing, China) for high-throughput sequencing and later analysis [19].

After high-throughput sequencing, the extracted genomic DNAs acted as templates to amplify the V3-V4 region of the 16S rDNA genes in each sample using 515F/806R primers (515F, 5′-GTGCCAGCMGCCGCGGTAA-3′; 806R, 5′-GGACTACVSGGGTATCTAAT-3′). The PCR products were analyzed with sequence reads on an Illumina HiSeq 2000 platform (Genebank accession number PRJNA560151).

### 2.4. Statistical Analysis

R platform (R Foundation for Statistical Computing, Vienna, Austria) was used in statistical analysis. Principal coordinate analysis (PCoA) and Partial Least Squares Discriminant Analysis (PLS-DA) were performed using the ‘ape’ package based on the UniFrac distances between samples. Metastats software (http://metastats.cbcb.umd.edu/) was used in the analysis of similarities and multi-response permutation planning methods to further assess the differences between groups. The Quantitative Insights into Microbial Ecology (QIIME) software package was processed to analyze the representative sequences of operational taxonomy units (OTUs) with their relative abundance, which were applied to calculate the rarefaction analysis and Shannon diversity index [20]. Sequences with ≥ 97% similarity were assigned to the same OTUs, which was performed with QIIME. The Kyoto Encyclopedia of Genes and Genomes (KEGG) resource (http://www.kegg.jp/ or http://www.genome.jp/kegg/) was carried out to evaluate the high-throughput sequencing data to get further metabolic function, and this KEGG disease database was investigated here.

In-house Perl scripts were used to analyze alpha (within samples) and beta (among samples) diversity. Data was presented as mean ± SD, and the statistical significance was set at *p* < 0.05 for correction of multiple comparisons [21].

## 3. Results

### 3.1. Compositions and Relative Abundance of Bacterial Communities in Water Samples

**Figure 2:**
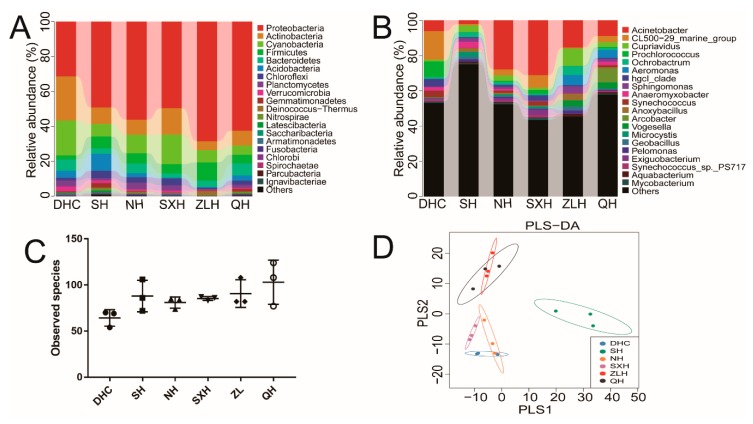
The diversity and richness of water bacteria among the Dahuchi (DHC), Shahu (SH), Nanhu (NH), Sixiahu (SXH), Zhelinhu (ZLH), and Qianhu (QH) groups. The relative abundance of the bacteria among these DHC, SH, NH, SXH, ZLH, and QH groups at the phylum level (**A**) and at the genus level (**B**). The x-axis represents the groups and the y-axis represents the relative abundance presented as a percentage. The amount of observed species in DHC, SH, NH, SXH, ZLH and QH groups (**C**). The x-axis shows the different groups and the y-axis shows the observed species. Validation of PLS-DA for species similarity and distribution among DHC, SH, NH, SXH, ZLH and QH groups (**D**).

The high-throughput sequencing was used to investigate the relative microbial abundance in the DHC, SH, NH, SXH, ZLH and QH waters. A total of 734,167 filtered clean reads (40,787.06 reads/sample) and 1537 OTUs were obtained from all of the samples with an average of 85.39 OTUs per group (data not shown). All the studied water samples showed high bacterial diversity (observed species from 64 to 103), and illustrated the significant differences in bacterial richness and diversity among these samples. At the phylum level, there were four most predominant phyla, including *Proteobacteria*, *Actinobacteria*, *Cyanobacteria* and *Firmicutes*, accounting for 78.9% in the DHC, 72.6% in the SH, 81.3% in the NH, 86.9% in the SXH, 91.0% in the ZLH and 81.1% in the QH. *Proteobacteria* was the largest bacterial phylum in all samples. Compared with other groups, *Acidobacteria* in SH was higher (9.6%) (Figure 2A). At genus level, dominant bacteria were identified from DHC [*Acinetobacter* (6.1%), *CL500-29_marine_group* (16.2%) and *Prochlorococcus* (8.9%)], SH [*Microcystis* (2.7%), *Anoxybacillus* (2.1%) and *Geobacillus* (1.4%)], NH [*Acinetobacter* (27.9%), *CL500-29_marine_group* (3.4%) and *Cupriavidus* (3.1%)], SXH [*Acinetobacter* (31.1%), *CL500-29_marine_group* (7.1%) and *Sphingomonas* (0.6%)], ZLH [*Acinetobacter* (15.4%), *Cupriavidus* (9.8%) and *Aeromonas* (5.8%)] and QH [*Acinetobacter* (8.9%), *Arcobacter* (7.8%) and *Aeromonas* (4.5%)], respectively.

In addition, our results indicated that the number of observed species in NH, SXH and ZLH groups were similar, the highest observed species was QH and the lowest observed species was DHC (Figure 2C). The result of PLS-DA illustrated that QH and ZLH gathered together, DHC, NH and SXH gathered together, and dots in SH scattered far away from other groups (Figure 2D).

### 3.2. Taxa Abundance of Bacterial Species and KEGG Analysis in Water Samples

**Figure 3:**
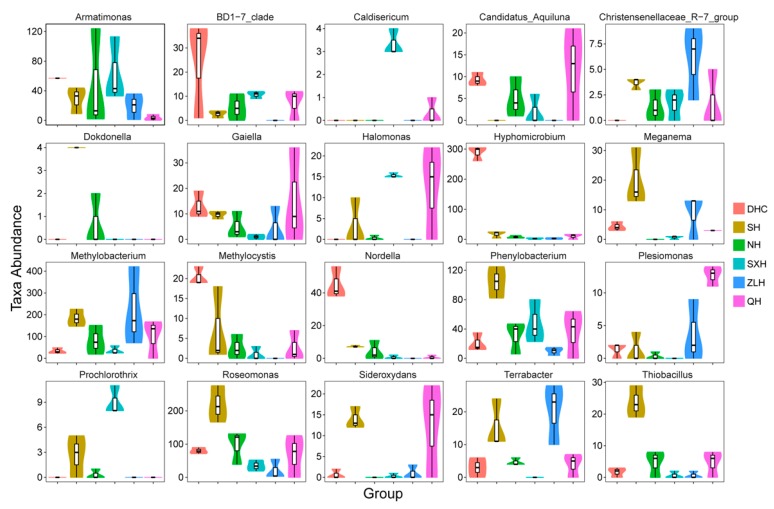
Taxa abundance of different bacteria species in groups DHC, SH, NH, SXH, ZLH and QH, which were sampled from their water.

We further studied the specific species in water of the DHC, SH, NH, SXH, ZLH and QH lakes, and found that DHC had the highest taxa abundance of *Armatimonas*, *BD1-7_clade*, *Gaiella*, *Hyphomicrobium*, *Methylocystis* and *Nordella*; SH had the highest abundance of *Dokdonella*, *Meganema*, *Methylobacterium*, *Phenylobacterium*, *Roseomonas*, and *Thiobacillus*; DHC and SH exhibited similar taxa abundance of *Plesiomonas*; NH and SXH had similar taxa abundance of *Armatimonas*, *Christensenellaceae_R-7_grou7p* and *Meganema*; SXH had the highest abundance of *Caldisericum* and *Prochlorothrix*; NH and QH, SXH and ZLH showed the similar *Thiobacillus* respectively; NH and ZLH showed the similar *Gaiella*; ZLH had the highest abundance of *Christensenellaceae_R-7_group* and *Terrabacter* and the QH had the highest abundance of *Halomonas*, *Plesiomonas* and *Sideroxydans* (Figure 3).

**Figure 4:**
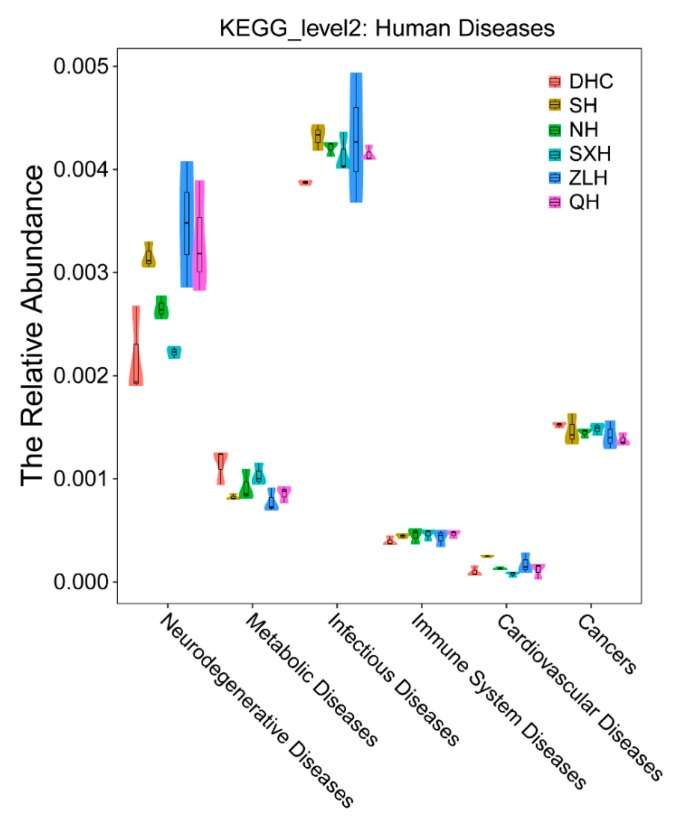
PICRUSt prediction of related human disease caused by DHC, SH, NH, SXH, ZLH and QH groups based on the Kyoto Encyclopedia of Genes and Genomes (KEGG) PATHWAY Database.

Then the KEGG analysis was used to predicate the potential effect of the bacteria in different lakes on human health (Figure 4). Our results indicated that more bacteria associated with neurodegenerative diseases had been observed in ZLH and QH, and more bacteria associated with infectious metabolic diseases were observed in DHC. In addition, the number of bacteria connected with infectious and cardiovascular diseases was upregulated in SH.

### 3.3. Compositions and Relative Abundance of Bacterial Communities in Sludge Samples

**Figure 5:**
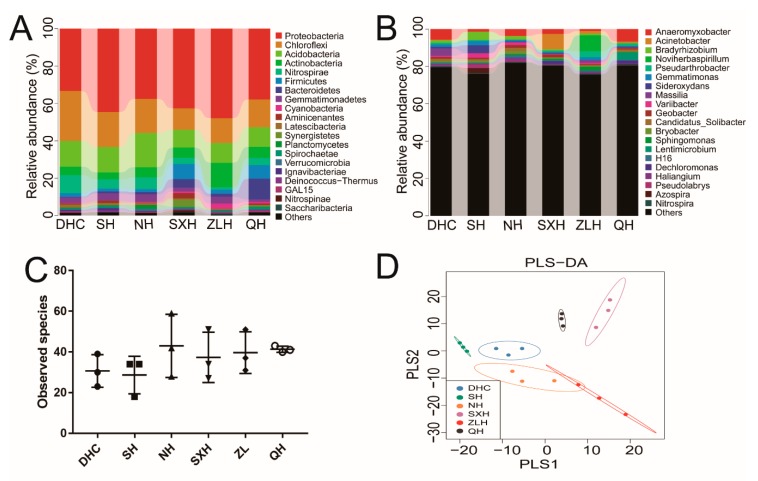
The diversity and richness of sludge bacteria among the DHC, SH, NH, SXH, ZLH and QH groups. The relative abundance of the bacteria among these DHC, SH, NH, SXH, ZLH, and QH groups at the phylum level (**A**) and genus level (**B**). The x-axis represents the groups and the y-axis represents the relative abundance presented as a percentage. The amount of observed species in the DHC, SH, NH, SXH, ZLH and QH groups (**C**). The x-axis shows the different groups and the y-axis shows the observed species. Validation of PLS-DA for species similarity and distribution among the DHC, SH, NH, SXH, ZLH and QH groups (**D**).

To better evaluate the microbial diversity, the microbial composition in sludge was also studied. A total of 805,345 filtered clean reads (4,4741.89 reads/sample) and 662 OTUs were obtained from all the samples with an average of 36.78 OTUs per group (data not shown). All the studied sludge samples illustrated the significant differences in bacterial richness and diversity among these samples. As demonstrated in Figure 5A, the dominant bacterial phylum in all groups was *Proteobacteria*, and the composition of *Chloroflexi* and *Acidobacteria* in DHC (26.7% and 13.8%), SH (18.7% and 13.6%), and NH (18.2% and 18.3%) were higher than that in SXH (11.4% and 9.6%), ZLH (13.2% and 10.6%), and QH (14.8% and 10.6%). The *Acitinobacteria* existed in the highest abundance in ZLH (13.0%), and the *Bacteroidetes* took the highest percentage in QH (11.0%). As shown in Figure 5B, the dominant bacterial genus of sludge samples in DHC, NH and QH was *Anaeromyxobacter*, accounting for 5.6% in DHC, 3.7% in NH and 6.7% in QH. The dominant bacterium in SH was *Bradyrhizobium*, accounting for 4.4%, and the dominant bacterium in SXH was *Acinetobacter*, accounting for 8.2%. The richness of *Noviherbaspirillum* in ZLH was obviously higher than others, accounting for 8.5% of the total number. Little difference of observed species was observed from DHC and SH, NH (43) and ZLH (28.7) showed the highest and lowest observed species, respectively (Figure 5C). The PLS-DA results indicated a relatively uniform bacterial diversity in all groups (Figure 5D).

### 3.4. Taxa Abundance of Bacterial Species in Sludge Samples

**Figure 6:**
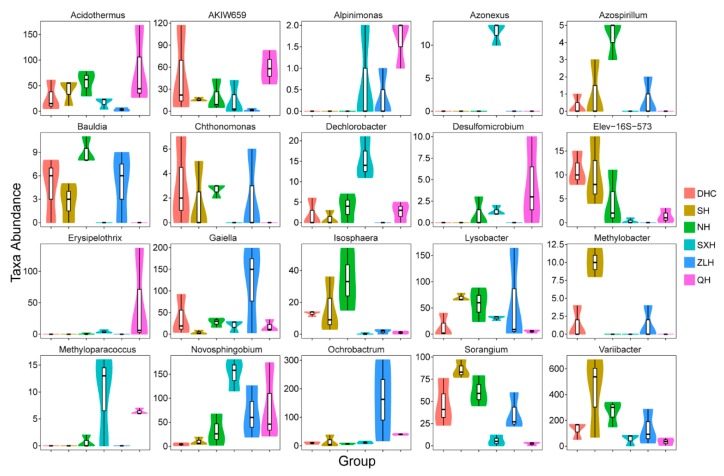
Taxa abundance of different bacteria species in groups DHC, SH, NH, SXH, ZLH and QH, which sampled from sludge.

The results of taxa abundance analysis illustrated that DHC had the highest taxa abundance of *Elev-16s-573* compared with the same taxa in other lakes (Figure 6); SH had the highest taxa abundance of *Lysobacter*, *Methylobacter*, *Sorangium* and *Variibacter*; NH had the highest abundance of *Acidothemus*, *Azospirilum*, *Bauldia*, *Chthonomas* and *Isosphaera*; DHC, SH and NH exhibited similar abundance of *Dechlorobacter*; SXH had the highest abundance of *Azonexus*, *Dechlorobacter*, *Methyloparacoccus* and *Novosphingobium*; The high abundance of *Azonexus* only existed in SXH; ZLH had the highest taxa *Gaiella* and *Ochrobactrum*, and QH had the highest taxa abundance of *AKIW659*, *Alpinimonas* and *Desulfomicrobium*. Only SXH, ZLH and QH had *Alpinimonas*, and QH had the highest taxa abundance. The abundance of *Novosphingobium* in order from lowest to highest was DHC, SH, NH, ZLH, QH and SXH.

## 4. Discussion

Poyang Lake has important regulation functions in the conserving watershed, recharging water from other rivers and discharging water to the Yangtze River [22]. In addition, the PLW, as one of the most important wetland nature reserves in the world, provides the main wintering habitat for the species in China, which can alleviate and adapt to the theoretical global climate change [23,24]. Thus it is necessary to pay more attention to the conditions of PLW, especially the water quality. As we know, the water quality can be easily influenced by natural processes (degrade surface waters) and anthropogenic factors (drinking, industry, agriculture, recreation and other purposes) [25], which brings water shortage and eutrophication, eventually causes toxic algal blooms, fish kills, loss of oxygen, biodiversity, aquatic plant beds and coral reefs [26,27]. Bacterial community is a bio-indicator for water quality and the degree of contamination, and it has a strong connection with nutrient cycling, energy flow, climate regulation, sediment accretion, pollutant filtration and biodiversity maintaining [28]. In this study, the obtained bacterial communities and related data using high-throughput sequencing suggested sharp differences in the bacterial composition among six studied lakes in both water and sludge samples.

As for water samples at the phylum level, *Actinobacteria* in water are widely distributed in both terrestrial and aquatic ecosystems and play an important role in the recycling of refractory biomaterials by decomposition and humus formation [29]. *Cyanobacteria* are essential geochemical agents and are regarded as key bacteria in carbon and nutrient cycles [30]. In Figure 2, a low abundance of *Actinobacteria* and *Cyanobacteria* were obtained in ZLH and QH, while a high proportion of these two phyla were observed in DHC. For DHC, it connects to the main lake area of Poyang Lake by adjacent rivers (Gan River and Xiu River) annually when the water level reaches 17.3 m, while artificial dams have been built in ZLH and QH, which cut off their connection to the main lake area of Poyang Lake. Therefore, the artificial construction and anthropogenic factors in ZLH and QH have reduced their capabilities on recycling refractory biomaterials, carbon and nutrients, compared with DHC.

At the genus level, *Acinetobacter* (*A. baumannii*) is a ubiquitous pathogen mainly found in pulmonary, urinary tract, bloodstream and surgical wound infections, having strong connection with antimicrobial resistance and health care-associated infections (HAIs) [31]. The bacterium *Aeromonas* is not only identified as an important disease-causing pathogen of fish and other cold-blooded species, but can also cause a variety of infectious complications in persons, such as septicemia and gastroenteritis [32]. In Figure 2, a highest number of *Acinetobacter* was observed in SXH and a high number of *Aeromonas* was observed in ZLH (artificial reservoir) and QH (urban lake), probably because of eutrophication induced by anthropogenic disturbance. In SXH, eutrophication can be caused by feeding, metabolites of fish and the fertilizing of nearby croplands. In ZLH, eutrophication may be caused by the activities of local residents. In QH, sewage injection is the major reason for causing eutrophication. Moreover, our KEGG analysis indicated that anthropogenic disturbances in ZLH and QH had greatly increased the bacterial number related with infectious diseases and neurodegenerative diseases (Figure 4).

In sludge samples, we found that a higher percentage of *Noviherbaspirillum* (usually find in oil contaminated sites) in ZLH, indicating the oil contamination by human activities in ZLH [33]. Similar with water samples, a high number of *Acinetobacter* had been observed from sludge samples in SXH, indicating human activities have increased the abundance of pathogens both in water and sludge. *Chloroflexi* always links to a dechlorination of chlorinated organic chemicals [34]. *Chloroflexi* has the ability to degrade soluble microbial products such as carbohydrates and can be applied to wastewater treatment and reclamation [35]. *Acidobacteria*, called green bacteria, can synthesize bacteriochlorophylls c, d or e and utilize chlorosomes for light harvesting [36]. The composition of *Chloroflexi* and *Acidobacteria* in DHC, SH and NH was higher than that in SXH, ZLH and QH, which means that SXH, ZLH and QH have a weaker ability of degradation and photosynthesis caused by anthropogenic disturbance, such as fishing, building dams and sewage disposal.

## 5. Conclusions

In the present study, our high-throughput sequencing results indicated that anthropogenic disturbance had greatly reduced the self-repairing capability of lakes, reduced the abundance of probiotic bacteria (*Actinobacteria*, *Cyanobacteria, Chloroflexi* and *Acidobacteria*) in lakes, and increased the abundance of bacteria which are harmful to lakes and humans (*Acinetobacter*, *Aeromonas* and *Noviherbaspirillum*). The present study researched for the detailed differences of bacterial communities in the water and sludge of six sub-lakes in PLW and analyzed the reasons for these differences. The results can provide positive and scientific data for ecosystem conservation and biological resources development, thus providing subsequent guidance for the management of the water quality of PLW, as well as provide reference for disease prevention of residents in the PLW.

## References

[1] Huang X., Hu B., Wang P., Chen X., Xu B. (2016). Microbial diversity in lake–river ecotone of Poyang Lake, China. Environ. Earth Sci..

[2] Chandrajith R.L.R., Okumura M., Hashitani H. (1995). Human influence on the Hg pollution in Lake Jinzai. Appl. Geochem..

[3] Wu L., Ge G., Zhu G., Gong S., Li S., Wan J. (2012). Diversity and composition of the bacterial community of Poyang Lake (China) as determined by 16S rRNA gene sequence analysis. World J. Microbiol. Biotechnol..

[4] Zhang Y.F., Tao C.Y., Huang Y. (2011). Study on Control Countermeasures of Agricultural Non-point Source Pollution in Lakeside Belt of Poyang Lake—Taking Duchang Section in the Lower Reaches of Poyang Lake as Example. Meteorol. Environ. Res..

[5] Quan W., Yan L. (2002). Effects of Agricultural Non-point Source Pollution on Eutrophica tion of Water Body and Its Control Measure. Acta Ecol. Sin..

[6] Nilsson C., Reidy C.A., Dynesius M., Revenga C. (2005). Fragmentation and flow regulation of the world’s large river systems. Science.

[7] Gutknecht J.L.M., Goodman R.M., Balser T.C. (2006). Linking soil process and microbial ecology in freshwater wetland ecosystems. Plant Soil.

[8] Liu X., Li Y.L., Liu B.G., Qian K.M., Chen Y.W., Gao J.F. (2016). Cyanobacteria in the complex river-connected Poyang Lake: Horizontal distribution and transport. Hydrobiologia.

[9] Lin Y.R., Du Y.P., Yang H.M. (2013). Analysis of wetland ecosystem response of Poyang Lake with water level evolution base on TM images. Appl. Mech. Mater..

[10] Mei X., Dai Z., Fagherazzi S., Chen J. (2016). Dramatic variations in emergent wetland area in China’s largest freshwater lake, Poyang Lake. Adv. Water Resour..

[11] Zhang B., Kang X. (1994). The features of the natural resources and the renovation strategy of Poyang Lake. Chin. Geogr. Sci..

[12] Shao M., Jiang J., Guo H., Zeng B. (2014). Abundance, distribution and diversity variations of wintering water birds in Poyang Lake, Jiangxi Province, China. Pakistan J. Zool.

[13] Venosa A.D., Lee K., Suidan M.T., Garcia-Blanco S., Cobanli S., Moteleb M., Haines J.R., Tremblay G., Hazelwood M. (2002). Bioremediation and Biorestoration of a Crube Oil-Contaminated Freshwater Wetland on the St.Lawrence River. Biorem. J..

[14] Lew S., Lew M., Mieszczyński T., Szarek J. (2010). Selected fluorescent techniques for identification of the physiological state of individual water and soil bacterial cells—Review. Folia Microbiol..

[15] Lew S., Glińska-Lewczuk K., Burandt P., Obolewski K., Goździejewska A., Lew M., Dunalska J. (2016). Impact of environmental factors on bacterial communities in floodplain lakes differed by hydrological connectivity. Limnologica.

[16] Ren Z., Gao H. (2019). Ecological networks reveal contrasting patterns of bacterial and fungal communities in glacier-fed streams in Central Asia. PeerJ.

[17] Kou W., Zhang J., Lu X., Ma Y., Mou X., Wu L. (2016). Identification of bacterial communities in sediments of Poyang Lake, the largest freshwater lake in China. SpringerPlus.

[18] Besemer K., Moeseneder M.M., Arrieta J.M., Herndl G.J., Peter P. (2005). Complexity of bacterial communities in a river-floodplain system (Danube, Austria). Microb. Ecol..

[19] Yu X., Wu X., Qiu X., Wang D., Gan M., Chen X., Wei H., Xu F. (2015). Analysis of the intestinal microbial community structure of healthy and long-living elderly residents in Gaotian Village of Liuyang City. Appl. Microbiol. Biotechnol..

[20] Caporaso J.G., Kuczynski J., Stombaugh J., Bittinger K., Bushman F.D., Costello E.K., Fierer N., Peña A.G., Goodrich J.K., Gordon J.I. (2010). QIIME allows analysis of high-throughput community sequencing data. Nat. Methods.

[21] Meng F., Chen T., Wang X., Wang X., Wei H., Tian P., Wang H., Zhao X., Shen L., Xin H. (2017). Evaluation of the accuracy and sensitivity of high-throughput sequencing technology using known microbiota. Mol. Med. Rep..

[22] Zhang W., Cao X., Peng J. (2008). Analyzing the 2007 drought of Poyang Lake Watershed with MODIS-derived Normalized Difference Water Deviation Index. Remote Sens. Model. Ecosyst. Sustainability V.

[23] Yan H., Zhan J., Jiang Q. (2010). Scenario simulation of changes of forest land in Poyang Lake watershed. Procedia Environ. Sci..

[24] Shao M., Zeng B., Tim H., Chen L., You C., Wang H., Dai N. (2012). Winter ecology and conservation threats of scaly-sided Merganser Mergus squamatus in Poyang Lake watershed, China. Pakistan J. Zool..

[25] Sánchez E., Colmenarejo M.F., Vicente J., Rubio A., García M.G., Travieso L., Borja R. (2007). Use of the water quality index and dissolved oxygen deficit as simple indicators of watersheds pollution. Ecol. Indic..

[26] Carpenter S.R., Caraco N.F., Correll D.L., Howarth R.W., Sharpley A.N., Smith V.H. (1998). Nonpoint pollution of surface waters with phosphorus and nitrogen. Ecol. Appl..

[27] Brown L.R., Halweil B.J. (1998). China’s water shortage could shake world food security. World Watch.

[28] Ansola G., Arroyo P., de Miera L.E.S. (2014). Characterisation of the soil bacterial community structure and composition of natural and constructed wetlands. Sci. Total Environ..

[29] Ventura M., Canchaya C., Tauch A., Chandra G., Fitzgerald G.F., Chater K.F., van Sinderen D. (2007). Genomics of actinobacteria: Tracing the evolutionary history of an ancient phylum. Microbiol. Mol. Biol. Rev..

[30] Bullerjahn G.S., Post A.F. (2014). Physiology and molecular biology of aquatic cyanobacteria. Front. Microbiol..

[31] Achenbach L.A., Michaelidou U., Bruce R.A., Fryman J., Coates J.D. (2001). Dechloromonas agitata gen. nov., sp. nov. and Dechlorosoma suillum gen. nov., sp. nov., two novel environmentally dominant (per)chlorate-reducing bacteria and their phylogenetic position. Int. J. Syst. Evol. Microbiol..

[32] Janda J.M., Abbott S.L. (2010). The genus Aeromonas: Taxonomy, pathogenicity, and infection. Clin. Microbiol. Rev..

[33] Lin S.-Y., Hameed A., Arun A.B., Liu Y.-C., Hsu Y.-H., Lai W.-A., Rekha P.D., Young C.-C. (2013). Description of Noviherbaspirillum malthae gen. nov. sp. nov. isolated from an oil contaminated soil, and proposal to reclassify Herbaspirillum soli, Herbaspirillum aurantiacum, Herbaspirillum canariense and Herbaspirillum psychrotolerans into the genus Nov. Int. J. Syst. Evol. Microbiol..

[34] Chen Y., Dai Y., Wang Y., Wu Z., Xie S., Liu Y. (2016). Distribution of bacterial communities across plateau freshwater lake and upslope soils. J. Environ. Sci..

[35] Miura Y., Watanabe Y., Okabe S. (2007). Significance of Chloroflexi in performance of submerged membrane bioreactors (MBR) treating municipal wastewater. Environ. Sci. Technol..

[36] Bryant D.A., Liu Z., Li T., Zhao F., Costas A.M.G., Klatt C.G., Ward D.M., Frigaard N.-U., Overmann J. (2012). Comparative and Functional Genomics of Anoxygenic Green Bacteria from the Taxa Chlorobi, Chloroflexi, and Acidobacteria. Funct. Genomics Evol. Photosynth..

